# The challenge of measuring mosquito flight performance: going beyond sterile insect technique and into transgenic and gene drive-based approaches

**DOI:** 10.1098/rsob.240400

**Published:** 2025-06-25

**Authors:** Paola Najera, Christian E. Ogaugwu, Tyler F. Chan, Raja Babu Singh Kushwah, Zach Adelman

**Affiliations:** ^1^ Texas A&M University, College Station, TX, USA; ^2^ Department of Entomology, Texas A&M University, College Station, TX, USA

**Keywords:** gene drive, sterile insect technique, flight assays, mark–release–recapture

## Abstract

Invasive insects inflict global costs of more than 70 billion USD annually by destroying crops and spreading disease-causing pathogens. Sterile insect technique (SIT), an insect population control method, involves the irradiation or chemical sterilization of insects to produce sterile males that are mass-released. SIT has proven effective in reducing populations of the Mediterranean fruit fly, Mexican fruit fly and screwworm fly. In the past decade, efforts to improve SIT with transgenic approaches have increased, including the development of potentially highly invasive gene drive transgenes. Determining flight capability is vital to the success of any insect control programme, and various flight assays can be used to analyse insect dispersal, flight behaviour and the mechanics behind flight. However, traditional flight assays such as mark–release–recapture become more challenging with transgenic or gene drive arthropods due to ecological concerns, while assays such as wind tunnels or flight mills/arenas may not capture the full range of flight abilities. This review seeks to cover current flight assays and their limitations as well as the requirements for flight assays to establish comparative flight ability for genetically modified insects to better prioritize strains prior to any potential field-based releases.

**Figure:**
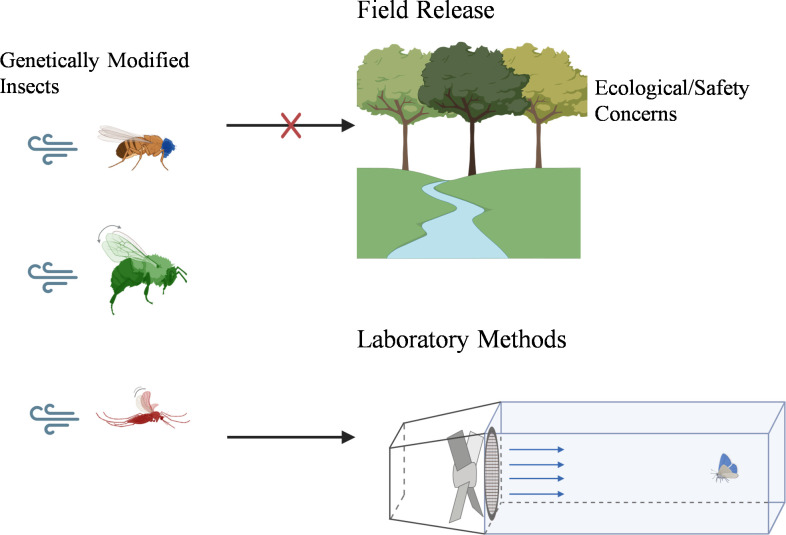

## 1. Introduction

Flight is an important aspect of the biology of many agricultural pests and disease vectors, as it directly affects their fitness. Insects that are good fliers usually have better survival than those that are poor fliers. Both basic and applied research have great interest in the flight abilities of insects because it gives a measure of their general quality [1], and their potential for dispersal or survival. Over years, the assessment of flight performance or capability has become a critical component necessary for the success of insect control programmes such as the sterile insect technique (SIT) and several population suppression or replacement strategies as it is imperative that insects utilized should disperse far enough to locate water, food, shelter and also integrate with the target population to achieve their main objectives [2,3].

The SIT is an insect population control method that employs infertile mating of released sterile males with wild females to reduce or suppress the wild population of the same species [4–6]. This method was first applied by Edward Knipling for the control of the screwworm fly *Cochliomyia hominivorax* [7,8]. SIT provides a species-specific alternative for insect control compared to chemical insecticides and other methods [9]. In practice, SIT involves the mass-rearing and mass-release of sterile males of the insect species desired to be controlled. Mass rearing can be done in small-, medium- or large-scale facilities, depending on what is feasible for the specific control programme. Sterilization is usually performed by exposure of the mass-reared pupae or adult insects to X-rays or ionizing gamma radiation [6,10]. Mass releases can then be carried out by delivery of pupae or adults to suitable locations on the ground or by air using aircrafts or uncrewed aerial vehicles, also known as drones [11,12]. SIT has been used successfully for more than seven decades to control various destructive or invasive insect pest species around the world such as the new world screwworm *C. hominivorax*, the tsetse fly *Glossina* spp., the pink bollworm *Pectinophora gossypiella*, the codling moth *Cydia pomonella*, the Oriental fruit fly *Bactrocera dorsalis*, the Mexican fruit fly *Anastrepha ludens*, the Mediterranean fruit fly *Ceratitis capitata*, the melon fly *Bactrocera cucurbitae* and others [13]. A pilot SIT study was carried out in Captiva Island, Florida in 2022 to assess relevant parameters for SIT control of *Aedes aegypti* [14]. Currently, SIT is being used in Fort Myers, Florida, USA in order to control the mosquito *Ae. aegypti*, which is responsible for the transmission of several tropical diseases such as dengue, yellow fever, chikungunya, West Nile fever and Zika [15]. Despite the utilization of SIT in various control programmes, the process of mass-rearing, sex separation (sexing) and mass-releasing can be expensive, laborious and time-consuming [11,16,17]. Consequently, subsequent research has focussed on optimizing and improving SIT to reduce the labour and operational costs and increase the efficacy of the programme.

## 2. Use and limitations of the mark–recapture method

Currently, most SIT programmes determine flight capability of insects using the mark–recapture method [18–21]. Traditionally, the mark–recapture method is a population size estimation strategy in which a portion of a target population is caught, then marked with fluorescent powders, paints or dyes [22–24] before being released back to the population. A second sample of the population is caught at a later time point, and the number of marked and unmarked individuals is used to estimate the size of the target population [18]. For SIT, the mark–recapture method has been adapted and involves marking the mass-reared males with a dye at the pupal or adult stage, releasing them at a specific location, and then using appropriate traps to recapture the released males from locations at predetermined distances from the initial release location [25]. From this, an assessment of the flight and dispersal capability of the released males can be determined. This strategy is currently termed ‘mark–release–recapture’ (MRR) and has been validated in *Aedes albopictus* for SIT application in *Aedes* mosquitoes [25].

One of the most important strengths of MRR, especially for pest control programmes, is the release of insects into outdoor environments, allowing for free flight. Temperature, humidity and precipitation all affect mosquito activity and dispersal [26]. Assessing dispersal in the same environment where sterile insects will eventually be mass-released provides a more realistic measure regarding the potential success of SIT programmes. MRR can also provide more insight into several parameters such as the size of the wild population, the relative field competitiveness of sterile insects, and average life expectancy of the released insects [27]. Despite the usefulness of the MRR method, it has several limitations. One disadvantage is that MRR relies on mass-rearing sterile insects, which can be expensive, time-consuming and even detrimental to dispersal. In one study, female *Culex tarsalis* that were reared in laboratory conditions dispersed significantly shorter distances than their wild trapped counterparts on average [28]. Another limitation of MRR is its use of markers. The dye applied as a mark on insects prior to the first release may wipe off or get transferred to other unmarked insects before they are recaptured later. In addition, dyes used for MRR can cause health problems and are expensive [29]. Additionally, many methods of marking require direct manipulation of individual insects, which may affect their flight. Vavassori *et al.* [30] sought to address this limitation by developing self-marking units that mark field mosquitoes with fluorescent pigment.

Within the past two decades, there has been increased development of transgenic or genetically modified (GM) insect strains into field programmes for control of disease vectors [31–34]. More recently, gene drives have been developed in disease vector insects [35–40]. Gene drive insect strains are also classified as GM insects but are unique because they have the potential to spread rapidly in the environment. While strict regulations guide the use of GM insects and the regulatory pathways may be much longer than for non-GM insects, GM insects have successfully been tested in the field and successfully used in field programmes for control of *Ae. aegypti* [41–44]. The MRR method was employed as an important component of the field tests of GM *Ae. aegypti* [41,45]. Notwithstanding, the MRR method would not be a suitable option for initial field tests involving gene drive insects as this will entail the releasing of the gene drive insects in the wild-pending recapture. Non-gene drive GM insect strains can disappear from the field within 2–5 months after stoppage of release [46], but gene drive insects have the capacity to rapidly spread and perpetuate in the environment. Consequently, a suitable, ethical, safe and yet rigorous method to test the flight capabilities of gene drive insects prior to their potential use in any control programmes such as SIT will be needed.

## 3. Measuring flight in laboratory assays

Besides MRR, several other laboratory and field-based flight assays have been developed that offer extensive insight into the flight behaviour of various insects, including many pest species. Some flight devices have also been used to observe a variety of behaviours, including oviposition, olfactory responses and dispersal. Examples of currently developed flight assays include wind tunnels, rotary flight mills, static tethered flight assays, flight arenas, wind tunnels and other flight testers (table 1). In this review, we aim to compare these flight assays, largely developed by experimental biologists studying insect behaviour, and evaluate their potential for use with transgenic insects developed by genetic engineers. Our hope is that by bringing these two communities together, their combined expertise can lead to the development of rigorous measurements of flight-based fitness in order to better prioritize transgenic strains for potential field-based evaluation.

**Table 1.** Summary of current flight assays.

flight assay	description	strengths	limitations	used for SIT?	used for transgenics?	pest species	ref.
static tethered flight	measures wing movement and force when insects are attached to a fixed point	simple to design and perform assays in the laboratory	may not reflect natural flight conditions	N	N	*Drosophila suzukii, Spodoptera litura*	[47,48]
rotary flight mill	measures distance and speed by tethering insects to rotating arms	simple to design and perform assays in a laboratory	can induce stress and unnatural flight behavior	Y	N	*D. suzukii,* *Ae. aegypti, Ae. japonicus, Amyelois transitella*	[47,49–51]
wind tunnel	uses controlled airflow to study free flight behaviour, distance, duration, velocity and periodicity in a contained environment	observes unrestrained flight in a controlled environment	complex set-up and can be expensive	Y	Y	*Ae. aegypti, Argyresthia conjugella, Lobesia botrana, A. transitella, D. melanogaster, Plutella xylostella*	[52–59]
flight arenas	uses video analysis to measure flight distance, duration, velocity and periodicity in a controlled environment	free flight assessment in a confined space	requires advanced analysis tools and expertise; stimulus to induce flight	Y	N	*Ae. aegypti, Ae. albopictus*	[60,61]
flight column	ability to fly from the point of release before hitting the ground	simple to design and perform the assays in the laboratory	focussed on a narrow aspect of flight: recovery	N	N	*D. melanogaster*	[62,63]
Flight Test Device (FTD)	escape rate from the flight device	flight evaluation under various stress conditions	requires advanced analysis tools and expertise	Y	N	*Ae. aegypti, Ae. albopictus, An. arabiensis*	[3,64,65]

### 3.1. Static tethered flight assays

**Figure 1.**
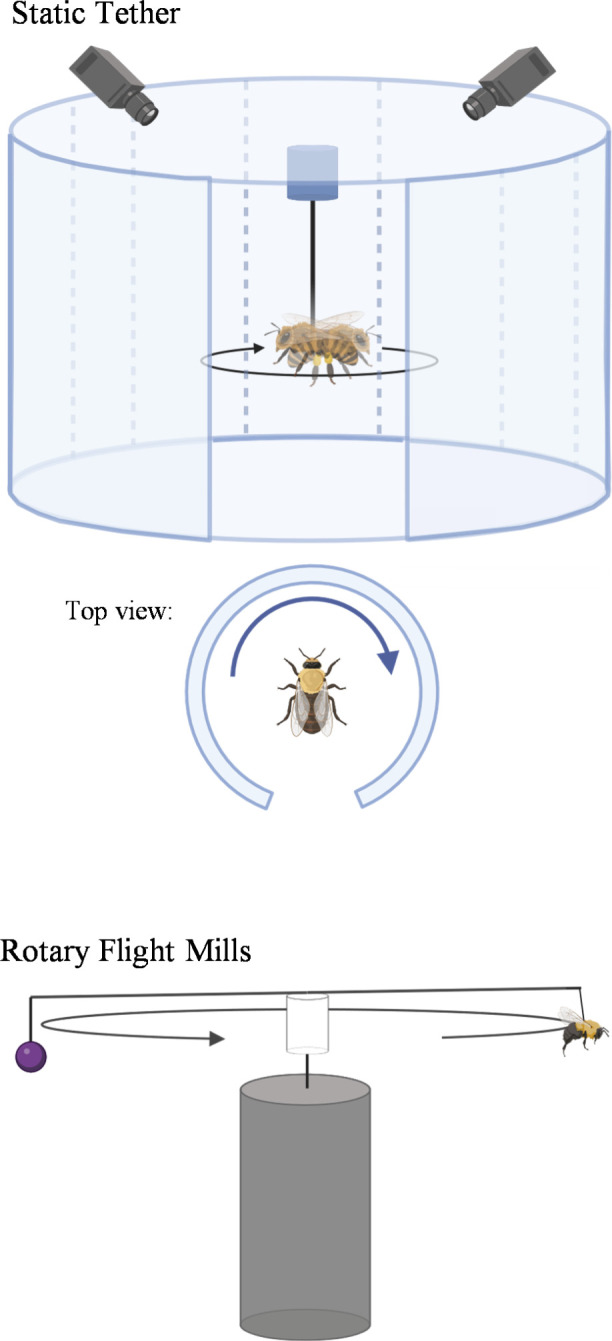
Tethered flight assays. Static tethers hold the insect, so the insect flies in place. The insect can rotate (in terms of pitch, yaw or roll) which can be measured using high-speed cameras. Flight mills use tethers to fix the insect to the mill. The insect can fly on a restricted flight path. Flight mills can measure velocity, distance flown and periodicity (the number of flight bouts initiated in a period of time). In both types of flight assays, the insect does not have to produce thrust since it is suspended in the air. Thus, results must be corrected for drag and lift.

Static tethering has been a common approach in insect behavioural studies. The insect is attached to a tether (such as wire) which is fixed, so when flight is induced, the insect flies in place (figure 1). Tethered flight assays can measure wing beat frequency; however, they cannot measure velocity since the insect is not changing position. Tethered flight assays can estimate flight duration and measure responses to changes in olfactory and visual stimuli.

In a study by Götz [66], fully fed tethered female *Drosophila melanogaster* could fly uninterrupted for up to 3 h. Static tethering has also been used to measure the effect of odours on wing beat frequency and amplitude [67]. A similar tethered flight assay measured changes in wing beat amplitude in response to visual stimuli [68]. Lawson & Srinivasan [69] tethered fruit flies in flight arenas to measure responses to changes in optic flow and wind speeds. These virtual-reality flight assays simulated movement and free flight for Queensland fruit flies (*Bactrocera tryoni*) while holding them in a flight arena. Changes in thrust and abdomen pitch were measured as responses to sinusoidal variation in optic and air flow. Four monitors on each wall of the arena were used to provide visual cues to simulate flight. A side camera was used to record a side-view of the fly to measure pitch. A recent study tethered Oriental fruit flies (*Bactrocera dorsalis*) and melon flies (*Zeugodacus cucurbitae*) used a vacuum to measure how long the fly can beat its wings without interruption [70].

A major advantage of static flight assays is their simplicity [71]. Static tethers are useful for measuring changes in pitch or yaw in insects in response to changes in wind speed. However, static tether flight assays have their limitations. Since the insect remains in one place, velocity and distance cannot be measured. Additionally, securing the insect onto the tether requires direct handling, potentially affecting fitness and limiting throughput.

### 3.2. Rotary flight mills

Flight mill studies require tethering an insect to the flight mill where it follows a fixed circular path (figure 1). Flight mills can measure velocity and flight distance since the insect is allowed to move (albeit on a restricted flight path) [71]. Counterweights are used to balance the flight mill and friction is minimized, facilitating flight in the tethered insect. Flight mills have been used in hundreds of studies with over 200 species spanning nine orders, especially Lepidoptera and Coleoptera [71]. Of these, Rowley & Graham [72] used flight mills on female *Ae. aegypti* and recorded flight distance, duration, speed and weight lost. The average distance flown by mosquitoes was 7459 m in the first week and 9108 m in the second week. In another study by Rowley & Graham [73], *Ae. aegypti* mosquitoes were flown to the point of exhaustion on flight mills at varying temperature and humidity levels. The longest distances flown were achieved at 21°C, regardless of humidity.

Flight mills could be used to estimate velocity, dispersal and flight capability in transgenic insects. Despite the relevance of flight mill studies, a major limitation is that the insect is not generating lift and is instead being held up by the tether. Consequently, it is unclear how flight performance on a flight mill will translate to the field. For example, in the emerald ash borer (*Agrilus planipennis*), the mean flight speed during free flight was more than three times higher than the mean flight mill speed [74]. Generally, heat production and metabolic rate reflect the effort made by an insect during sustained flight. Previous literature shows that insects held up by a tether have a reduced temperature and metabolic rate, such as bumblebees which have approximately half the metabolic rate when flying with a support [75,76]. Future research should work to further quantify the difference between flight mill flight and free flight. Additionally, flight mills could be improved so that energy expenditure of tethered insects more closely resembles that of free-flying insects. Another constraint of fight mills is that the insect is limited to a specific flight path around the rotor. Consequently, the ability of a transgenic insect to ‘steer’ or manoeuvre in response to wind would not be elucidated using a flight mill. Finally, flight mill assays require direct handling of an insect, which may affect the flight performance. Overall, while flight mills may provide insight into the flight performance of transgenic and gene drive insects, they only provide part of the picture.

### 3.3. Wind tunnels

**Figure 2.**
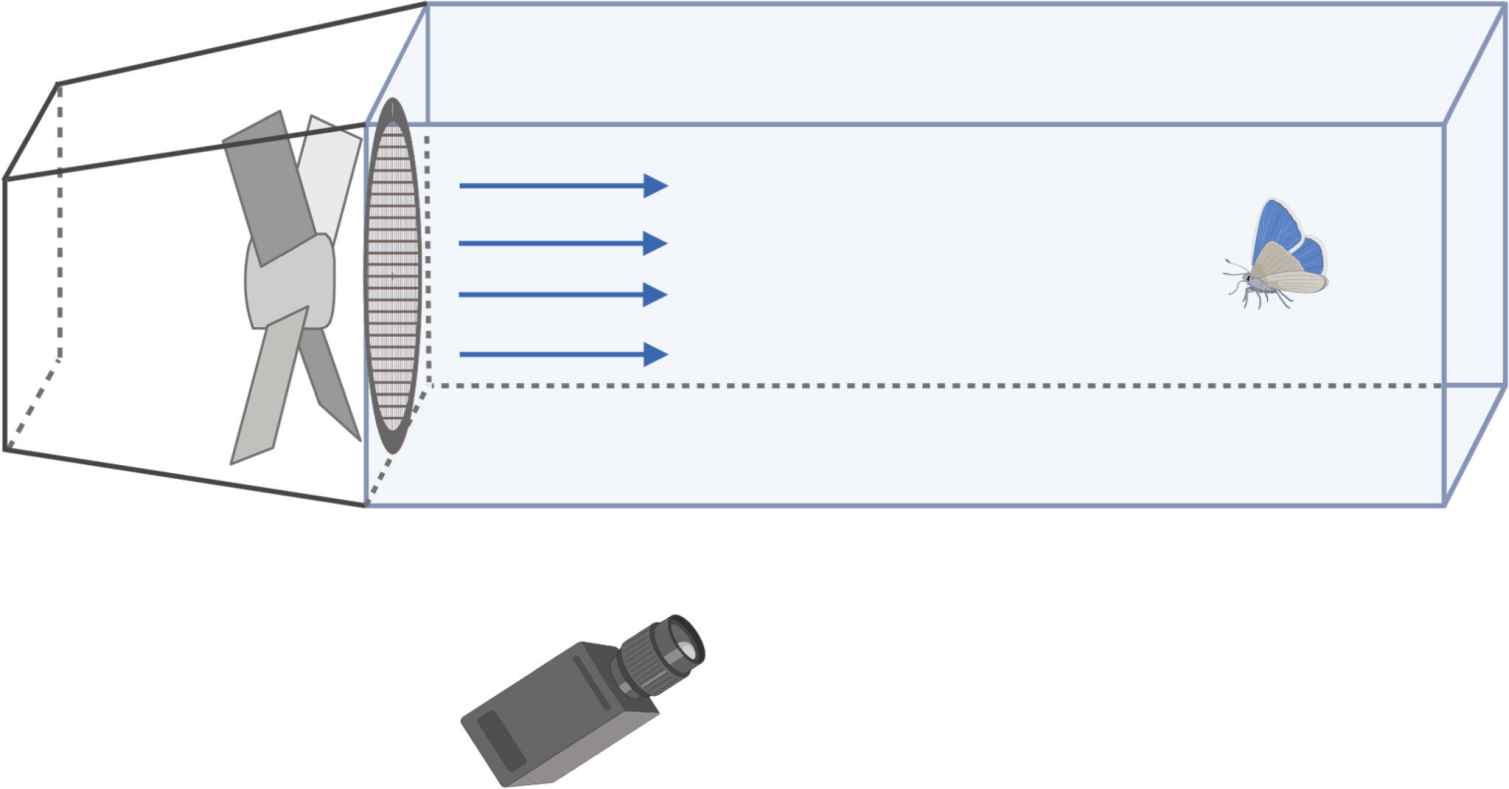
Wind tunnel. Wind tunnels are a commonly used behavioural assay used for olfactory and/or flight studies. The basic design of wind tunnels uses a tunnel where the insects are released, a fan to produce wind, mesh to provide laminar flow and a cue to induce flight in the insects. Wind tunnels can measure velocity, flight distance, flight duration and periodicity. They are often used in olfactory studies to observe flight behaviour in response to semiochemicals.

Wind tunnels use fans to stimulate outdoor wind conditions in a contained setting and allow for controlled experiments and direct observation of insect behaviour (figure 2). Wind tunnels have become a common tool in insect behavioural studies, where they are used to test responses to olfactory, visual and thermal stimuli or to focus on the mechanics responsible for flight in insects. Hinterwirth *et al.* [77] used electrical stimulation to manipulate the flight pitch of the hawkmoth (*Manduca sexta*), which uses its antennae to control its flight. Fine electrodes were inserted near each antennal rim on the hawkmoths’ head capsules. Synchronized high-speed cameras recorded the moths’ flight patterns through a wind tunnel. Altitude, velocity and body angle (in terms of pitch and yaw) were recorded for each moth using footage and individual frames. Combes & Dudley [78] explored the effect of aerial turbulence on the flight performance of male orchid bees (*Euglossa imperialis*). An artificial olfactory cue was placed in front of an air jet sourced from a wind tunnel. The free flight of the bees was recorded on high-speed cameras to determine their maximum speeds. The flow variability in front of the air jet was also measured. Air velocity was increased until the bees spun out of the jet stream.

More commonly, wind tunnel studies do not focus specifically on flight performance and instead are commonly used to test responses to semiochemicals and other environmental variables. For example, one study used a wind tunnel to test responses to environmental changes in 21 butterfly species [79]. The wind tunnel measured species-specific temperature preferences and feeder colour preference. Miller & Roelofs [80] constructed a wind tunnel to study the sustained flight of male red-banded leafroller moths (*Argyrotaenia velutinana*) in response to sex pheromones. The tunnel featured a continuous belt that moved in the same direction as wind with adjustable speeds ranging from 2 to 60 m min^−1^. Fouad *et al.* [81] used a wind tunnel to investigate the responses of the Angoumois grain moth (*Sitotroga cerealella*) to corn kernel volatiles. Responses were measured in males and females using the electroantennography (EAD) technique. Another study used a laminar flow wind tunnel to observe female *Ostrinia nubilalis* (European corn borer) flight behaviours [82]. Behaviours and their latency were scored over the course of 5 min in the presence of a maize plant. The behaviours included wing fanning, taking flight, upwind flight and landing. Behavioural scores were determined based on the frequency of behavioural steps.

Notably, many wind tunnel studies use larger insects (especially the order Lepidoptera). For example, the adult tobacco hawkmoth (*M. sexta*) has an average wingspan of 9.5–12 cm. However, (modified) wind tunnels are also useful for studying smaller insects such as mosquitoes. For example, Eiras & Jepson [52] used a wind tunnel equipped with two filters to measure take-off, flight, landing and probing responses to lactic acid, carbon dioxide and/or human sweat in female *Ae. aegypti*. Knols *et al.* [83] utilized a similar approach to test responses to volatile chemical stimuli in *Anopheles gambiae*. Two traps were placed in a wind tunnel, each of which had a test odour pumped through it, allowing mosquitoes to choose between the traps. Spitzen *et al.* [84] used a wind tunnel to study the changes in flight behaviour of *A. gambiae* (*s.s*.) in response to odour and heat. The 1.60-m wind tunnel was equipped with a computer-controlled air treatment system that provided wind gusts with a speed of 0.20 m s^−1^. Two monochrome video cameras were used to record the infrared light reflected off of the mosquitoes’ wings.

Wind tunnels allow for free flight, providing a potentially more realistic estimation of flight performance in the field, while still securely containing the insects. Unlike flight assays which require tethers, wind tunnels allow researchers to observe whether insects can lift off of the ground or walls. Additionally, by containing the insects, wind tunnels hold promise for use with transgenic insects, particularly gene drive strains, since they can be used before approval for field release is granted. However, many wind tunnel studies have required the use of high-speed cameras, requiring specialized expertise/equipment. Second, typically only one insect can enter the tunnel at a time, making data generation a relatively slow and labour-intensive process. Overall, wind tunnel studies have offered insight into insect behaviour and hold promise for use with transgenic insects; progress in simplifying image acquisition and flight tracking (discussed below) may make these assays more accessible to labs developing and testing transgenic insects.

### 3.4. Flight arenas

**Figure 3.**
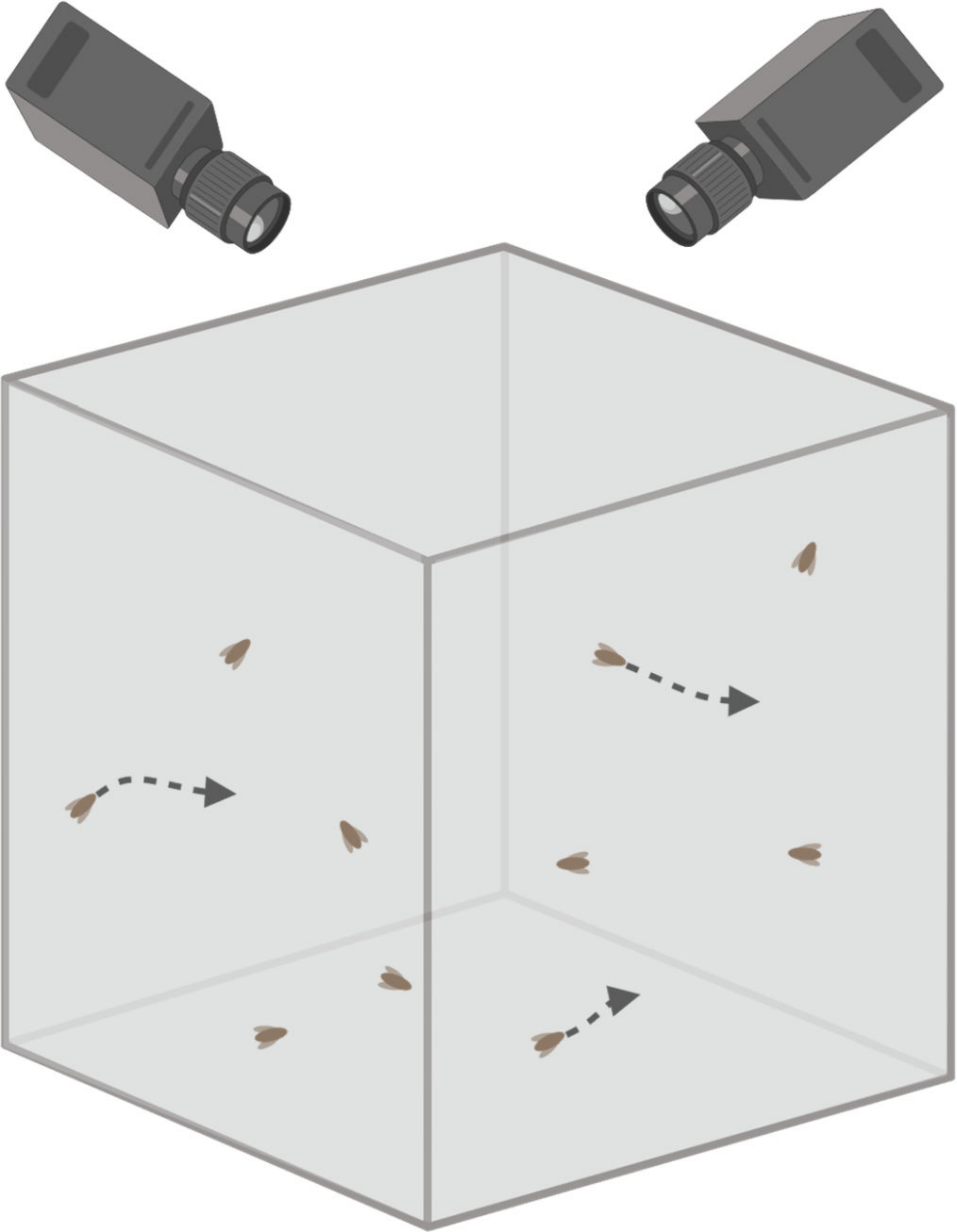
Flight arena. Typically cubes, flight arenas allow for free flight, which is tracked using video cameras. A cue is needed to induce flight. Flight arenas can be used to measure flight distance, duration, velocity and periodicity.

The phrase ‘flight arena’ has been used to describe a wide variety of flight assays. Here, we define flight arenas as chambers where insects are allowed to fly freely (figure 3). Flight arenas tend to be cubes that are at least a metre in length and width. High-speed cameras are necessary to track the flight paths of each individual insect and estimate its velocity.

Bomphrey *et al.* [85] developed a flight arena for blowflies using mirrors to create a corner-cube reflector. A high-speed camera was used to measure flight performance, using various parameters including velocity, acceleration and turning. Additionally, the flight trajectories of the blowflies were plotted using automated tracking. Several blowflies were released into the flight arena at a time, but no more than 14 flies, and individual identities could not be determined. Another flight arena was later designed that used a novel tracking system capable of tracking swarms of fruit flies (*D. melanogaster*) at a time; this arena was much smaller with side lengths only 36 cm long [86].

FlyCave is a metre-high cylindrical flight arena that was used with *D. melanogaster* [87]. The FlyCave used FreemoVR, a VR system designed for free-moving animals, to project realistic visual stimuli onto the walls of the flight arena. Tracking software and cameras were used to track the flies' flight paths.

A major strength of flight arenas is allowing for free flight while still securely holding the insects, meaning they hold potential for testing gene drive insects. Additionally, flight arenas do not require direct handling to attach a tether like flight mills or static tether flight assays. However, a stimulus is still needed to induce flight. Flight arenas also require (multiple) high-speed cameras to track the movement of the free-flying insects; insect flight tracking is discussed in more detail later in this review. Flight arenas also do not typically incorporate environmental factors such as wind or humidity. Nevertheless, flight arenas could be used to test the flight performance of transgenic insects. Improved flight arena designs that could better evaluate responses to changes in wind, temperature or humidity would be of benefit to evaluating flight capabilities and fitness of transgenic/gene drive insects.

### 3.5. Other flight testers

Many novel flight assays that have been designed to assess the flight performance of insects are not easily categorized. Nevertheless, the following flight assays hold promise for use with transgenic insects, including those in gene drive systems. Here, we group these flight assays to evaluate their strengths and limitations. Since these flight assays have been recently developed, there is limited literature on their use with insects, especially transgenic insects.

### 3.6. Flight column

**Figure 4.**
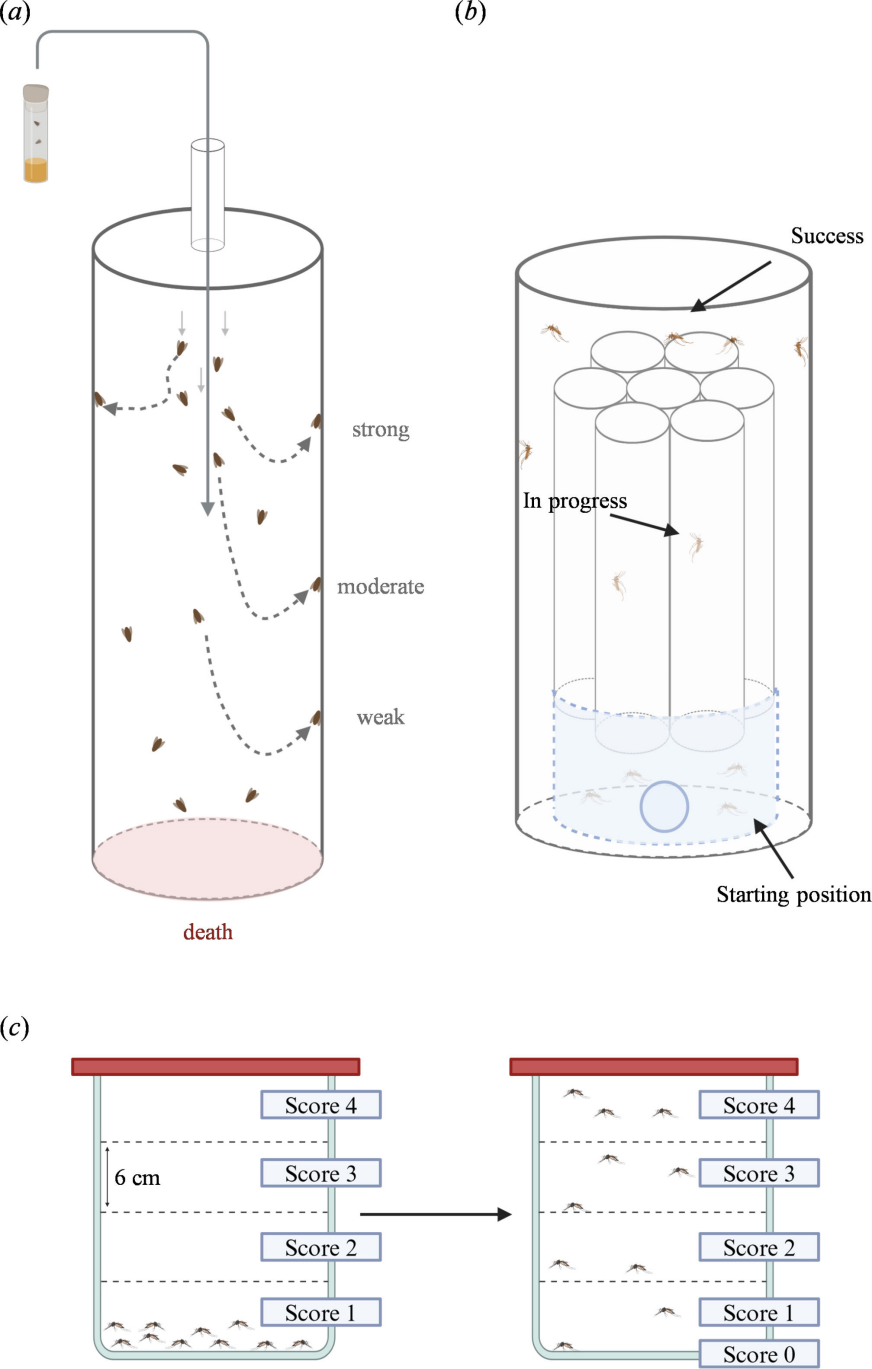
Other flight testers. (*a*) *D. melanogaster* are dropped into the metre-high flight column where they fall freely. The flies recover from falling and land at varying heights of the column. Based on their landing position, the flies are categorized as weak, moderate or strong fliers. Flies that could not recover fall into mineral oil at the bottom of the column. (*b*) The flight test device (FTD) was designed to evaluate the flight performance of mosquitoes before mass-release for insect control programmes. Mosquitoes are placed into the FTD, where they must fly up and escape through one of the flight tubes. Successful mosquitoes can be collected from the larger containment tube. (*c*) The induced flight activity test (INFLATE) quantifies flight activity in mosquitoes. The cage is tapped on a benchtop to gather the mosquitoes on the bottom of the cage. After 1 min, the mosquitoes are scored based on their position in the cage, with a score of zero assigned to the mosquitoes still on the bottom of the cage. The flight cage is divided into four quadrants with scores ranging from one to four as height increases.

Babcock & Ganetzky [63] developed a flight tester which involved dropping *D. melanogaster* into a graduated cylinder with an oil-covered base (figure 4*a*). Flight performance was assessed by measuring how far the flies fell before recovering and landing on the walls of the cylinder. Flies that could not produce enough thrust to land onto the cylinder walls fell into mineral oil underneath the flight cylinder, permanently removing them from the flight assay. An improved version included a drop tube to control for initial velocity and adhesive insect traps on the inside walls of the column, allowing for the visualization of the differences in flight capabilities. A separate study used this flight column with high-speed cameras to compare nearly 200 *D. melanogaster* Genetic Reference Panel (DGRP) lines [62]. The average landing height of the flies following the sudden drop were used to categorize the various DGRP lines as having strong, intermediate or weak recoveries.

A flight column measures recovery in insect flight, which may aid survival in the field. While it does not include environmental factors, such as wind, in its evaluation of flight ability, this flight assay could be used with others to evaluate the flight performance of transgenic, specifically gene drive, insects.

### 3.7. Dispersal flight cages

Other flight assays have used oviposition to evaluate the dispersal of female mosquitoes. Reiter *et al.* [88] used rubidium-marked eggs to estimate the dispersal of female *Ae. aegypti* when laying eggs. They learned that ovipositing females lay their eggs at various sites over the course of several days, covering an area approximately 840 m in diameter. Brown *et al.* [89] used 30 m linear cages to study whether the availability of potential oviposition sites influences dispersal in female gravid *Ae. aegypti*. Each flight cage was constructed using a PVC frame and fibreglass insect screen with oviposition containers placed along the flight cage (sparsely or densely depending on treatment). After one week, recording how many eggs were found in each container gave an approximation of how far the gravid females travelled. These flight assays could be considered to develop a flight assay that allows gene drive insects to disperse in the environment, while remaining contained.

### 3.8. Flight test device

Culbert *et al.* [64] developed a FTD to assess the flight capability of male mosquitoes with SIT applications in mind. The FTD consists of 40 clear acrylic flight tubes inside a larger polymethyl methacrylate (PMAA) tube (figure 4*b*). Up to 100 *Ae. aegypti* or *Ae. albopictus* mosquitoes were loaded into the confined base of the FTD underneath the tubes and allowed to fly untethered for 2 h. The mosquitoes can then fly up through one of the 40 tubes which are 0.25 m long. The mosquitoes that escape the flight tubes enter the larger holding tube where they can be scored. Maïga *et al.* [65] optimized the original FTD to produce consistent and reproducible results for quality control (QC). They explained the factors affecting device performance for quality control of sterile mosquitoes, emphasizing the importance of evaluating flight performance under different stress conditions. Specifically, they measured the effects of cold stress and high-dose irradiation (conditions common to SIT insects) on success rate. The study also measured the effects of parameters including fan speed, mosquito strain, mosquito age and density on success rate. Fan speed and mosquito strain, for example, did not appear to affect the success rate in *Ae. albopictus*. Mosquito age was correlated with significant differences in success rate. For example, *Ae. aegypti* mosquitoes that were older than 4 days performed at a significantly higher rate than those younger than 3 days. Male density did not significantly affect success rate in *Ae. aegypti*, but a lower density resulted in a higher success rate in *Ae. albopictus*. The suggested protocol for the FTD was to run each assay for 120 min, using a transparent middle flight tube, a fan and a BG lure for each mosquito age group. Further research should assess the effects of temperature, humidity and light [65]. Similar standards should be developed for transgenic insects to optimize flight assays to ensure consistent quality. A major barrier to the widespread use of the FTD was the price. However, the optimized version allows for a fraction of the inner tubes, allowing for a more cost-effective device without sacrificing consistent results.

### 3.9. Induced flight activity test

The INduced FLight Activity TEst (INFLATE) was developed to induce and quantify flight activity in *Ae. aegypti* [90]. The INFLATE assay used a 24 cm tall flight cage, which is divided into four horizontal quadrants 6 cm in height) and holds 10 mosquitoes at a time (figure 4*c*). The flight cage is tapped on a benchtop to gather the mosquitoes on the bottom. After 1 min, the mosquitoes were counted based on their location to calculate an INFLATE score, with higher quadrants associated with higher scores. After repeating 19 times, the median score was used to determine an INFLATE index. The INFLATE is simple to replicate and easy to use to quantify flight performance. While it may not measure the environmental effects on flight, its design is highly accessible and reproducible. Overall, the INFLATE would not provide a full picture of the flight performance of gene drive insects in the field but could be of use due to its reproducibility.

## 4. Application of machine vision and deep learning to flight assays

Many of the flight performance assays described rely on machine vision for quantification, which relies on computerized collection and analysis of video/photo data. Mechanical tools such as flight mills can pose challenges in interpretation due to confounding effects of insect mounting procedure, resistive forces and presence/absence of natural flight cues may cause the arthropod flight performance data to differ from flight performance measured in a field setting [91]. In contrast, imaging data can quantify unencumbered flight characteristics of mosquitoes or other insect species and can also monitor the movement of objects in three dimensions. However, collecting three-dimensional position data requires at least two imaging devices and an even larger number of stills to digitize from multiple perspectives, increasing the data processing workload; a report of capture methods by Poh *et al.* [92] reported several studies of insect flight recording and analysis that produced large amounts of data, as much as 1 terabyte of data per hour of recording.

### 4.1. High-contrast insect flight tracking

More affordable and higher quality computational power and digital cameras have assisted the development of machine vision tracking, which can perform automatic detection and positioning of various moving objects (e.g. vehicles, particles, insects) against a contrasting backdrop [93–96] using image processing algorithms to identify objects and then analyse their trajectory. In a laboratory setting, the position of insects can be determined by using backdrops and carefully selected lighting that provides very high contrast between airborne objects and background, and the positions of single insects over time can be determined by numerous free and commercial software [96–102]. Shakeel *et al.* [103] reviewed existing insect tracking techniques in further detail.

### 4.2. Measured parameters

Using single or multiple-object contrast-based tracking has been used to quantify flight characteristics of wingbeat frequency [104], as well as position, velocity and acceleration [92,96]. Other studies using manual annotation of flight data have quantified other flight parameters such as orientation, rotational inertia and collision performance [105], as well as fluid flow surrounding mosquitos in-flight [106], though the data collected required manual human annotation and have yet to be transferred to automated approaches for mosquitos and other flying arthropods.

### 4.3. Machine learning and machine vision

Further developments in the field of machine learning have demonstrated the feasibility of using convolutional neural networks (CNNs) to identify, locate and track multiple insects and species against more complex backgrounds [107]. These neural networks, which can be tuned and configured for picking out mobile or stationary objects of interest against challenging backdrops, such as those with moving, heterogeneous or variable conditions [108], can provide more flexible and robust insect tracking software solutions. These neural network-based machine vision tools have been used to track the flight path of caged mosquitos [102], identify insect species in fielded camera traps [109,110], and identify the species of arthropod present in video analysis by using wingbeat frequency [104,107].

### 4.4. Desired machine vision characteristics of flight assays

As highlighted by Poh *et al.* [92], many existing captive insect flight capture imaging set-ups are ‘high-complexity’ in terms of imaging sensors, lenses, lighting equipment, cages and fixturing devices. The current field of machine vision insect flight quantification appears to be lacking in solutions that are simultaneously simple to set up, have reasonably attainable computational processing and data storage requirements, a flight tracking area of sufficient size and flexibility to suit experimenters’ needs, multi-object tracking capabilities, adaptability to heterogeneous (‘field condition’) backgrounds, and are adaptable to different species. These parameters are covered to varying degrees of satisfaction by existing machine vision flight assays, and new hardware, software and analysis packages are often developed from the ground up for new organisms and assays. Despite the ubiquity and continued refinement of both imaging equipment and open-source computer-vision modules, a one-size-fits-all fix has yet to be widely adopted for the diversity of species and flight parameters to quantify.

## 5. Future directions

SIT has been used as an approach to vector control since the late 1950s. The first release of sterile mosquitoes using SIT took place in Florida in 1959 [111]. While SIT is well-established and can cause rapid population suppression, it can prove expensive in the long term [112]. Typically, MRR has been used to test the dispersal of mosquitoes before an SIT programme [14]. However, this approach may not be appropriate for initial testing of the flight capabilities of gene drive mosquitoes, since the transgene may spread away from the release site and persist in the environment.

Flight assays for gene drive mosquitoes should be secure, allowing free flight without allowing the mosquitoes to access people or the environment. Additionally, assays should allow measurements of how far the mosquitoes can fly, how fast and whether they can withstand environmental factors (i.e. humidity, wind, freefall).

The aforementioned flight assays vary in their suitability for testing the flight performance of transgenic insects. While most flight assays have not been used with transgenic insects (table 1), specifically, wind tunnels and flight arenas, which allow for free flight, may be ideal. Important advances needed would be to incorporate environmental conditions in their designs, while reducing the cost (simplified motion tracking) and increasing throughput (moving from single insect to swarm-based assays). Other assays such as flight mills and static tethering are both simpler to implement, but require direct handling, which may damage insects and may not provide a full picture of a mosquito’s flight performance due to limitations in flight path. The flight column and dispersal cages both provide limited but important aspects of flight ability and may be good complementary assays. The FTD holds promise for use with transgenic mosquitoes but again, could be improved to include environmental factors such as temperature and humidity.

## Data Availability

This article has no additional data.
